# Biological Efficacy of Compounds from Stingless Honey and Sting Honey against Two Pathogenic Bacteria: An In Vitro and In Silico Study

**DOI:** 10.3390/molecules27196536

**Published:** 2022-10-03

**Authors:** Shirmin Islam, Mohammad Joy Pramanik, Suvro Biswas, Mohammad Moniruzzaman, Jui Biswas, Mohammad Akhtar-E-Ekram, Shahriar Zaman, Mohammad Salah Uddin, Mohammad Abu Saleh, Sabry Hassan

**Affiliations:** 1 Microbiology Laboratory, Department of Genetic Engineering and Biotechnology, University of Rajshahi, Rajshahi 6205, Bangladesh; 2 Department of Biology, College of Science, Taif University, P.O. Box 11099, Taif 21944, Saudi Arabia

**Keywords:** honey, *Bacillus cereus*, *Listeria monocytogenes*, antibacterial activity, molecular docking, molecular dynamics simulation

## Abstract

Honey inhibits bacterial growth due to the high sugar concentration, hydrogen peroxide generation, and proteinaceous compounds present in it. In this study, the antibacterial activity of stingless and sting honey against foodborne pathogenic bacteria isolated from spoiled milk samples was examined. The isolated bacterial strains were confirmed as *Bacillus cereus* and *Listeria* *monocytogenes* through morphological, biochemical, and 16 s RNA analysis. Physiochemical characterizations of the honey samples revealed that both of the honey samples had an acidic pH, low water content, moderate reducing sugar content, and higher proline content. Through the disc diffusion method, the antibacterial activities of the samples were assayed and better results were observed for the 50 mg/disc honey. Both stingless and sting honey showed the most positive efficacy against *Bacillus cereus.* Therefore, an in silico study was conducted against this bacterium with some common compounds of honey. From several retrieved constituents of stingless and sting honey, 2,4-dihydroxy-2,5-dimethyl 3(2H)-furan-3-one (furan) and 4H-pyran-4-one,2,3-dihydro of both samples and beta.-D-glucopyranose from the stingless revealed high ligand-protein binding efficiencies for the target protein (6d5z, hemolysin II). The root-mean-square deviation, solvent-accessible surface area, the radius of gyration, root-mean-square fluctuations, and hydrogen bonds were used to ensure the binding stability of the docked complexes in the atomistic simulation and confirmed their stability. The combined effort of wet and dry lab-based work support, to some extent, that the antimicrobial properties of honey have great potential for application in medicine as well as in the food industries.

## 1. Introduction 

An antimicrobial substance either eliminates or prevents the growth of bacteria. The bacteria that act largely against antibiotics can be used to classify antimicrobial medical drugs. Antibacterials, for instance, are used to treat bacteria, and antifungals for fungi. It is now well-acknowledged that honey contains antibacterial properties, and these properties depend on several different mechanisms of action [1]. The global issue of antibiotic resistance has made the quest for novel antibacterial compounds extremely important [2]. The emergence of bacteria that are resistant to antibiotics and can cause foodborne illnesses as well as other diseases has been linked to antibiotic use. On the other hand, market trends in the food industry are ever-evolving. Nowadays, we seek higher-quality foods, which means fewer extreme treatments and/or additives, more recently developed and organic products, and wholesome diets [3]. It is important to use as few antibiotics as possible when producing food, especially organic food. The antibacterial properties of organic honey might offer a way to preserve organic produce.

Honey has been used in traditional medicine for ages [4]. Over 200 distinct elements make up the intricate chemical structure of honey. About 75% of honey’s constituents are monosaccharides, with the remaining 15% being water. Additionally, the composition of honey includes trace amounts of organic acids, flavonoids, minerals, and wax particles. Compound constitutions vary slightly as a result of regional influences and environmental factors [5,6]. The compounds of honey are mostly from the group of flavonoids, furan, and glycol. However, the basic compositions of the honey remain more or less the same [7,8]. Numerous in vitro studies have demonstrated that a particular honey has antimicrobial activity against a variety of bacteria that colonize the skin and cause foodborne illnesses including bacteria that are resistant to antibiotics [9,10,11,12,13]. According to Robson et al. [14], honey has been demonstrated to be effective against wound infection in vivo. Honey products that have been approved for use in wound care are also frequently used [15]. Recently, the significant antibacterial efficacy of modified honey against wound infections has been revealed. It is made out of honey that has undergone processing to produce different levels of antibacterial activity. Additionally, some honey components have been identified through in silico research to be useful against several serious illnesses such as SARS-CoV-2 [16,17].

The main antibacterial components are hydrogen peroxide, low pH, and high osmolality [18,19]. Phenolic chemicals may potentially enhance the effectiveness of antibiotics [20]. Revamil^®^ and Manuka honey, the two medical honey varieties most frequently used in wound therapy, both offer additional antibacterial properties. Methylglyoxal is the primary active ingredient in Manuka honey [21], while bee defensin-1, an antibacterial peptide, has been identified in Revamil^®^ honey [12].

Numerous investigations on honey’s antibacterial action have been carried out in non-European nations, particularly in the Southern Hemisphere [22,23]. There have been many clinical study on the use of New Zealand Manuka honey. However, additional types of honey have been found with comparable inhibitory action and floral backgrounds [6]. It is recognized that the foodborne pathogen can cause a wide range of illnesses in both humans and animals. It is one of the leading causes of foodborne illness in the USA, Europe, Japan, and Asia [24].

Therefore, this study concentrated on isolating pathogenic bacteria from samples of spoiled milk and identifying honey’s antibacterial activities against the foodborne pathogenic bacteria. Additionally, conducting an in silico study to uncover a unique compound that would be capable of eradicating and introducing antibacterial agents against foodborne pathogenic microorganisms. 

## 2. Materials and Methods 

Locally available stingless honey and sting honey samples were collected from the Saheb Bazar grocery shop, Rajshahi, and the milk sample was collected from Rajshahi University campus. 

### 2.1. Isolation of Pathogenic Bacteria from Milk 

A total of 100 μL of spoiled milk was taken in the liquid medium with a micropipette on the laminar airflow bench and incubated for 16 to 18 h at a temperature 37 °C with shaking at 60 rpm to prepare the bacterial mixed culture. The serial dilution method was used to select a single colony from the mixed culture. MacConkey agar media were used for the isolation of pathogenic enteric bacteria. The single colony was selected and streaked several times for a pure bacterial colony. A pure single colony was transferred into LB liquid medium for storage and further use. 

### 2.2. Identification through the Morphological, Biochemical Test, and Molecular Technique

Morphological and biochemical tests were performed for the specific identification of bacteria. Bacterial isolates were characterized by several morphological and biochemical tests such as Gram staining, motility, urease, catalase, methyl red, indole, mannitol, starch hydrolysis, triple sugar iron (TSI), citrate, and the eosin methylene blue (EMB) agar test.

For the species identification of the isolates, 16S rRNA genes were sequenced from Invent technology and compared with other sequences from the gene bank database using the Basic Local Alignment Search Tool (BLAST) available from the website (www.ncbi.nlm.nih.gov/Blast, accessed on 15 February 2022) [25].

### 2.3. Role of pH and Temperature on Bacterial Growth

To determine the optimum pH for bacterial growth, the culture medium was adjusted to a pH ranging from 3.5 to 8.5 with 0.5 intervals. Data were recorded at 25 °C, 30 °C, 33 °C, 35 °C, 37 °C, 40 °C, and 45 °C after 12 h of incubation. OD was measured at 600 nm with a UV–Vis spectrophotometer (Analytik Gena, Thuringen, Germany). 

### 2.4. Antibiotic Sensitivity Test of Isolated Bacteria 

Different antibiotics such as penicillin G, ampicillin, amoxicillin, ciprofloxacin, chloramphenicol, erythromycin, gentamycin, and tetracycline were used for an antibiotic sensitivity test. Discs were placed carefully on the respective plates and incubated overnight at 37 °C. After overnight incubation, the zone was observed on the plate and measured with the help of a mm scale. Gentamycin was used as the control. 

### 2.5. Physicochemical Analysis of Honey 

Water and sugar contents, pH, hydroxy methyl furfural, and proline are the major concerns of honey [26,27]. Thus, every sample should undergo some physiochemical analysis. Before starting the experiment, the following parameters were measured.

#### 2.5.1. pH Measurement

A total of 10 g of honey was mixed with 75 mL of distilled water [27] to measure the pH of honey with a pH meter. 

#### 2.5.2. Water Content

Using a refractometer, the water content of the honey was measured by the refractive index at 20 °C regarding the Chataway table [28].

#### 2.5.3. Hydroxymethyl Furfural (HMF)

HMF is produced by the degradation of fructose and glucose by intramolecular dehydration [29]. HMF is used to control the freshness and quality of honey. A value greater than 60 mg/kg indicates aging honey [30]. This was measured using Winkler’s method [31]. Data were measured at 550 nm in the presence of barbituric acid and para-toluidine by a UV-VIS spectrophotometer. 

#### 2.5.4. Total Sugars

Total sugars represent the dry matter of honey. Analysis was performed by the refractometer using the methods described by Helrich [27].

#### 2.5.5. Reducing Sugars

The amount of total reducing sugars (%) from the total sugars was determined by using the volumetric method [27].

#### 2.5.6. Proline Content

The proline content (mg/kg) was measured using a colorimetric assay with ninhydrin according to the Ough (1969) [32] methods as described by Bogdanov et al. (2002) [27], as it provides information about the maturity of honey and can also be used to detect forgeries. Honey is considered mature when the proline level is greater than 183 mg/kg, while lower values signify immaturity or fabrication [33].

### 2.6. Antimicrobial Test against the Isolated Pathogen 

The disc diffusion method was used in the current experiment to evaluate the antimicrobial activity of the selected honey. The discs (6 mm diameters) were made by punching Whatman No. 1 filter paper with the help of a punch machine. These discs were taken into the screw-capped tube and sterilized in an autoclave machine at 121 °C for 20 min to ensure sterilization. The paper discs were soaked with different concentrations (50, 100, 125, and 150 mg/disc) of honey suspension with the help of a micropipette and kept in a laminar airflow hood for dryness (5–10 min). The discs contain samples as well as the standard antibiotic discs (gentamicin 10 µg).

### 2.7. Assessment of Antimicrobial Activity 

The antimicrobial activity of honey was analyzed using a disc diffusion assay according to the technique described by Bauer et al. (1966) [34] and adapted by Taormina et al. (2001) [35]. The LB agar plate was prepared and 100 μL of bacterial culture was spread by a sterile spreader. Discs with different concentrations of honey were added to spreading plates with a control (here, gentamicin was used). After overnight incubation, the zones were observed and measured with a mm scale. 

### 2.8. In Silico Experiment

#### 2.8.1. Protein Preparation 

BIOVIA Discovery Studio Visualizer v.4.5 (Accelrys) was used to remove all water molecules and hetero-atoms from the three-dimensional crystal structure of the hemolysin II (HlyII) protein (https://www.rcsb.org/, accessed on 15 February 2022) (PDB ID: 6d5z), which was retrieved from the RCSB Protein Data Bank (http://www.pdb.org, accessed on 15 February 2022). AutoDock Tools v. 1.5.6rc3 was subsequently used to prepare a PDBQT file of the target protein containing added polar hydrogen atoms. 

#### 2.8.2. Ligand Preparation 

The compounds previously reported in different kinds of honey were selected as ligands for the docking experiment [36] and the SDF format of the 3D structures was retrieved from the PubChem database (http://www.pubchem.ncbi.nlm.nih.gov/ accessed on 15 February 2022) [37]. The mmff94 force field from Avogadro software was used to construct and improve the structures of the ligands [38].

#### 2.8.3. Molecular Docking

Molecular docking of all of the identified compounds was performed using the AutoDock Vina software tool. The ligands were then converted to PDBQT format, with the box size and grid box center set to (X: –0.310 Å, Y: −0.1525 Å, and Z: −0.0632 Å) and (X: −47.4831 Å, Y: −26.7732 Å, and Z: −27.6943 Å), respectively. The total number of steps, update steps, and energy difference for the ligand molecules were adjusted to 200, 1, and 0.1, respectively, utilizing the universal force field with conjugate gradient algorithms. The grid box dimensions were selected and set up to wrap the protein’s substrate-binding region. Auto Dock Vina’s performance was visualized using the DS visualizer program [39,40].

### 2.9. Molecular Dynamics Simulation

In YASARA software package version 20.1.1. (YASARA Biosciences GmbH, Vienna, Austria) [41,42], with the facilitation of the AMBER14 force field, the molecular dynamics simulation was performed [43,44]. Initially, the docked complexes were optimized and cleaned, and hydrogen bond interactions were oriented. As the cubic simulation cell was constructed using a periodic boundary condition, the TIP3P solvation model was applied [45,46]. Additionally, the simulation cell was extended by 20 Å in both directions apart from the complexes of the ligand and protein. The simulation cell’s physiological condition was set as 298 K, pH 7.4, and 0.9 percent NaCl. With the simulated annealing method, the elementary energy minimization was accomplished with the steepest gradient algorithms (5000 cycles). The simulation cell’s time step was set as 1.25 fs. The long-range electrostatic interactions were enumerated by a cut-off radius of 8.0 Å with the particle mesh Ewald system [47,48,49,50]. The simulation trajectories were stored after every 100 ps. Using a Berendsen thermostat along with constant pressure and temperature, the simulation was directed for 100 ns. To analyze the root mean square fluctuation (RMSF), the radius of gyration (Rg), root mean square deviation (RMSD), solvent accessible surface area (SASA), and hydrogen bond, the simulation trajectory data were utilized [51,52,53,54,55,56,57].

### 2.10. ADMET Analysis

The Swiss ADME (http://www.swissadme.ch/ accessed on 15 February 2022) [58] and pkCSM (http://biosig.unimelb.edu.au/pkcsm/ accessed on 15 February 2022) [59] tools were used for the evaluation of the pharmacological properties of ligands where canonical SMILES of the compound were used as the entry system for the absorption, distribution, metabolism, and toxicity (ADMET) calculations.

## 3. Results 

### 3.1. Isolation of Bacterial Strains on Selective Media 

**Figure 1:**
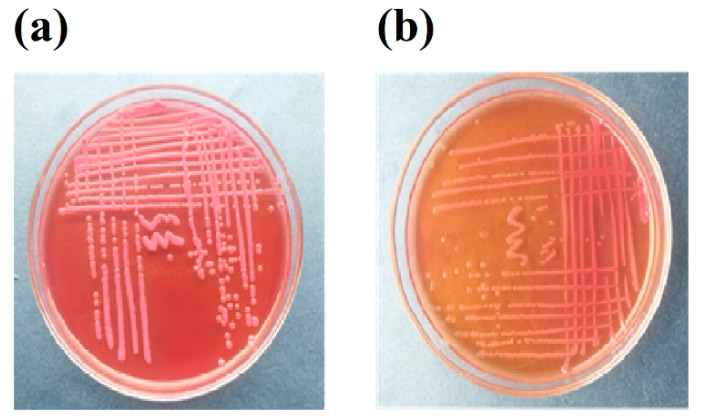
Streaking of Isolate A (**a**) and Isolate B (**b**) on MacConkey agar.

MacConkey agar medium was used for the isolation of bacteria from the spoiled milk (Figure 1). Isolate A and Isolate B were selected based on their morphological nature 

### 3.2. Identification of Bacterial Strains

In Gram staining, Isolate A and Isolate B showed rod-shaped purple-colored indicating Gram-positive (Table 1). Both the isolates were motile. Biochemical tests indicated that Isolate A and Isolate B were negative in the indole and mannitol agar tests. Isolate A was positive for the urease hydrolysis test, TSI, citrate, catalase, and starch hydrolysis tests whereas Isolate B was positive for the methyl red, catalase, and EMB tests (Table 1) (Figure S1).

From the 16S rRNA gene sequence analysis, it was found that Isolate A had 99.00% similarity with *Bacillus cereus* and Isolate B had 99.00% similarity with *Listeria monocytogenes*. Thus, Isolate A and Isolate B were confirmed as *Bacillus cereus* and *Listeria monocytogenes,* respectively.

### 3.3. Antibiotic Sensitivity Test

**Figure 2:**
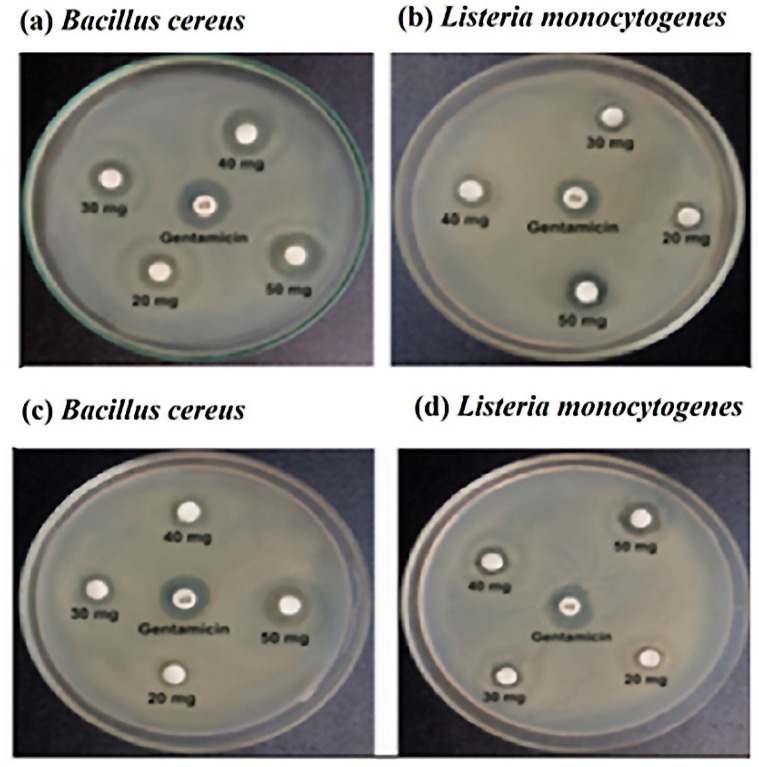
The zone of inhibition with stingless honey sample (**a**,**b**) and sting honey sample (**c**,**d**) against the *Bacillus cereus* and *Listeria monocytogenes,* respectively.

Various antibiotics were used in this experiment. Figure 2 and Table 2 showed that *Bacillus cereus* was resistant to penicillin G, and ampicillin and susceptible to ciprofloxacin, chloramphenicol, erythromycin, gentamycin, and tetracycline. On the other hand, *Listeria monocytogenes* was susceptible to amoxicillin, intermediate resistance to tetracycline, and resistance to penicillin G, ampicillin, ciprofloxacin, chloramphenicol, erythromycin, and gentamycin. 

### 3.4. Growth Characteristics

**Figure 3:**
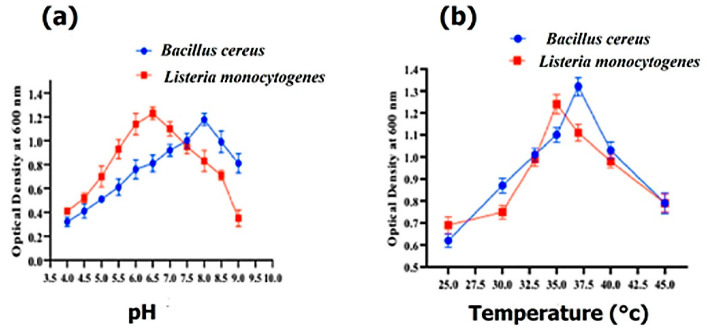
The effect of pH on the growth of *Bacillus cereus* and *Listeria monocytogenes* (**a**) and the effect of temperature (°C) on the growth of *Bacillus cereus* and *Listeria monocytogenes* (**b**).

The optimal growth of *Bacillus cereus* and *Listeria monocytogenes* was determined with various pH ranges from 3.5 to 8.5. *Bacillus cereus* and *Listeria monocytogenes* exhibited their maximum growth at pH 8.0 and 6.5, respectively (Figure 3a), whereas *Bacillus cereus* and *Listeria monocytogenes* showed their maximum growth at 37 °C and 35 °C, respectively (Figure 3b).

### 3.5. Physiochemical Characteristics of Honey Samples 

Results of the physicochemical analysis of the honey samples are shown in Table 3. From the table, it is clear that all of the samples contained between 13 and 16% water with an acidic pH (4.33–4.56). The hydroxymethyl furfural content varied from 71 to 123 mg/kg. The total sugars were above 80% in both samples, whereas the reducing sugars ranged from 67.84 to 77.10%. A higher proline concentration (833.02 ± 2.89 mg/kg) was found in the stingless honey.

### 3.6. Antibacterial Activity of Honey Samples against Bacillus Cereus and Listeria Monocytogenes

Both the sting and stingless honey demonstrated antimicrobial effects on specific bacterial strains. As the control, gentamicin (10 μg) was employed. For *Bacillus cereus*, stingless honey with concentrations of 20, 30, 40, and 50 mg/disc revealed inhibitory zones of 10 mm, 11 mm, 13 mm, and 14 mm, respectively, while the inhibitory zones for sting honey were 7.5 mm, 9 mm, 9.5 mm, and 12 mm, respectively. In the same concentrations, stingless honey demonstrated inhibition zones of 7.5 mm, 8.5 mm, 10 mm, and 12 mm against *Listeria monocytogenes*, while sting honey demonstrated inhibition zones of 7 mm, 8 mm, 9.5 mm, and 11 mm, against the same bacterial strain, respectively.

### 3.7. Molecular Docking of Honey Constituents against Hemolysin II (Protein) of Bacillus Cerecesus

**Figure 4:**
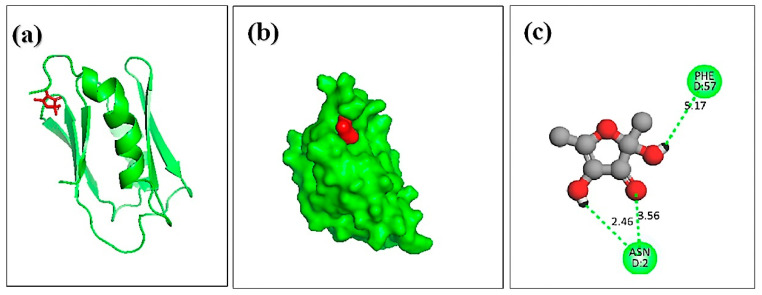
Docking simulation between the spike receptor-binding domain of hemolysin II (protein) and 2,4-dihydroxy-2,5-dimethyl 3(2H)-furan-3-one (furan), where (**a**) shows the cartoon view, (**b**) surface view, and (**c**) 2D view.

**Figure 5:**
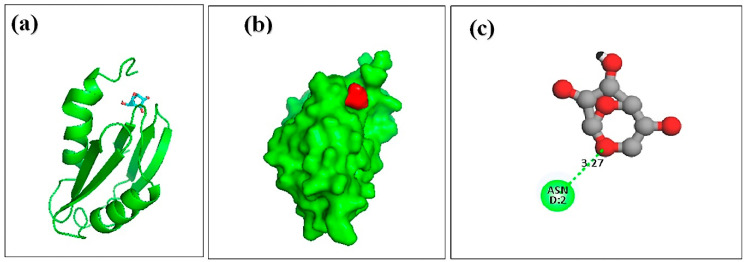
Docking simulation between the spike receptor-binding domain of hemolysin II (protein) and beta.-D-glucopyranose,1,6-anhydro, where (**a**) shows the cartoon view, (**b**) surface view, and (**c**) 2D view.

**Figure 6:**
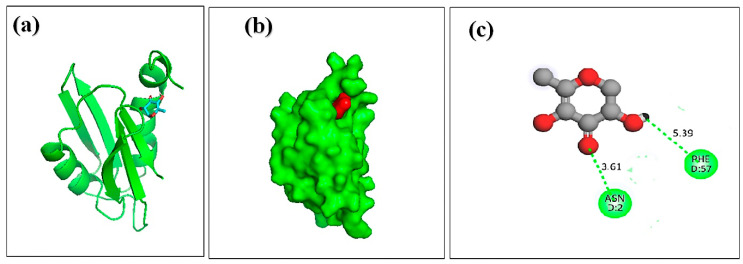
Docking simulation between spike receptor-binding domain of Hemolysin II (protein) and l 4H-Pyran-4-one,2,3-dihydro 3,5-dihydroxy-6-methyl, where (**a**) shows the cartoon view, (**b**) sur face view, and (**c**) 2D view.

Molecular docking analysis was carried out to explore the binding interaction and identify the lead molecules with a higher affinity for the hemolysin II protein of *Bacillus Cerecesus*. Twelve compounds of stingless honey and seven of sting honey from the previous papers were docked with the protein 6d5z (Tables S1 and S2). The top three compounds, 4-dihydroxy-2,5-dimethyl 3(2H)-furan-3-one (furan), beta-D-glucopyranose,1,6-anhydro, and 4H-pyran-4-one,2,3-dihydro3,5-dihydroxy-6-methyl- had binding energies of −5.4, −5.2, −4.8 Kcal/mol, respectively (Table 4). The 2,4-dihydroxy-2,5-dimethyl 3(2H)-furan-3-one (furan) and hemolysin II protein domain-receptor complexes were stabilized by two hydrogen bonds at ASN-2 and one and PHE-57 (Table 5, Figure 4); the beta.-D-glucopyranose,1,6-anhydro hemolysin II protein complex had one ASN-2 hydrogen bond (Table 5, Figure 5) and 4H-pyran-4-one, 2, 3-dihydro 3, 5-dihydroxy-6-methyl-hemolysin II protein complex had two hydrogen bonds at ASN-2 and PHE-57 (Table 5, Figure 6). 

### 3.8. Molecular Dynamics Simulation

**Figure 7:**
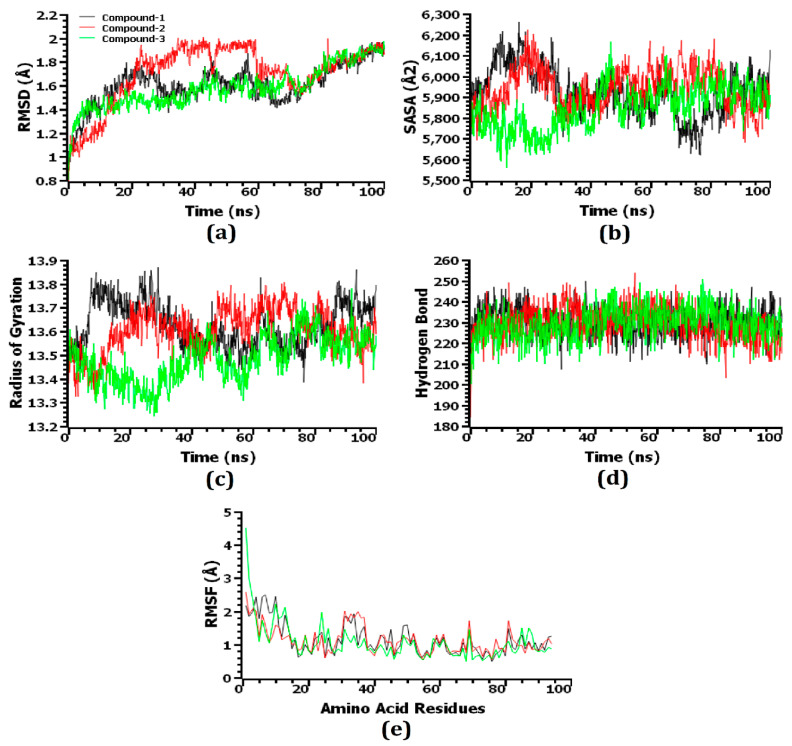
The simulated systems were analyzed on a time series basis. From (**a**–**e**), the RMSD of alpha carbon atoms is denoted by (**a**), protein volume with the expansion is denoted by (**b**), rigidity and compactness of the complexes are represented by (**c**), hydrogen bonding is represented by (**d**), and the flexibility of amino acid residues is denoted by (**e**).

Through the use of molecular dynamics simulations, the structural stiffness of the top three protein–ligand complexes was examined, and the docking possibilities for these complexes were verified. The RMSD of the C-alpha atoms was enumerated to explain changes in the stability of the protein–ligand complexes from the simulated trajectories. Due to their instability, complexes comprising compound 1 [2,4-dihydroxy-2,5-dimethyl 3(2H)-furan-3-one (furan)], compound 2 [beta.-D-glucopyranose,1,6-anhydro], and compound 3 [4H-pyran-4-one,2,3-dihydro3,5-dihydroxy-6-methyl-] displayed an initial increase in the RMSD value, as shown in Figure 7a. Compared to the three complexes, compound 2-hemolysin II exhibited the largest average increase in RMSD value. After roughly 65 ns, the RMSD value of the compound 1-hemolysin II complex dropped drastically, then it stabilized at around 80 ns and remained steady for the final 20 ns of the simulation time with very slight fluctuations. On the other hand, compared to the other two complexes, the compound 3-hemolysin II complex had a lower average RMSD value. The compound 2-hemolysin II complex displayed a somewhat greater RMSD value at 20–70 ns compared to the other two complexes, thereby explaining their enhanced flexibility. All three complexes exhibited some upward and inward RMSD trend before 80 ns of the simulation time, but they persisted steadily for the final 20 ns with only minor fluctuations. However, all three complexes exhibited an RMSD profile below 2.5 Å, representing that their stability was maintained over the lifetime of the simulation [56]. The SASA of the complexes was also evaluated to gain a deeper understanding of how the surface area of hemolysin II protein varies with time after coming into contact with the ligand molecules. The increasing value of SASA depicts the expansion of the surface area of the protein, whereas the decreasing value indicates the truncation of the surface area [56]. The compound 2-hemolysin II complex’s SASA was higher at a 50–85 ns simulation time than the other two complexes, signifying that it had an expanded surface area compared to the others (Figure 7b). The compound 1-hemolysin II and compound 2-hemolysin II complex displayed an initial increase in the SASA value, whereas the compound 3-hemolysin II complex showed a preliminary decrease in the SASA value. The compound 1-hemolysin II, compound 2-hemolysin II, and compound 3-hemolysin II complexes reached a steady state at the 80 ns, 30 ns, and 50 ns simulation times, respectively, and they persisted steadily for the remaining simulation time with only minor fluctuations. 

In order to determine whether the protein complexes were more compact or flexible, Rg values were used. Simulated protein complexes with a greater Rg value are considered more flexible, while those with a lower value are considered firmer [44]. The compound 1-hemolysin II and compound 2-hemolysin II complex exhibited a preliminary increase in the Rg value, which specifies their increased flexibility, whereas the compound 3-hemolysin II complex showed an initial decrease in the Rg value, which represents their higher stiffness (Figure 7c). The compound 1-hemolysin II complex displayed the highest Rg value during the 0–35 ns and 85–100 ns time frames whereas the compound 3-hemolysin II complex exhibited the lowest Rg value at the 10–75 ns and 80–100 ns simulation times with slight fluctuations.

As hydrogen bonds play a crucial role in the stability and integrity of proteins, the potential hydrogen bond formation was examined in the docked complexes. An extensive network of hydrogen bonds was formed in the compound 1-hemolysin II, compound 2-hemolysin II, and compound 3-hemolysin II complex throughout the entire simulation trajectory, which was indicative that the best three ligand molecules developed a tight connection with the hemolysin II protein (Figure 7d). An investigation of the RMSF of the top three ligands and hemolysin II complexes was conducted to further recognize the protein’s ability across the amino acid residues. A few amino acid residues in the compound 3-hemolysin II complex had RMSF profiles above 3 Å at the beginning, whereas every other residue in the top three docked complexes had RMSF profiles below 3 Å (Figure 7e). Based on their lower RMSF values, the top three docked complexes demonstrated less flexibility, which is consistent with their higher stability as lower RMSF values indicate a more stable complex [56].

### 3.9. ADMET Analysis

The drug-likeness of the top docked compounds is often predicted using the ADMET prediction. All of the chemicals complied with the Lipinski rule of five in the ADMET calculations (Table 6). All of the compounds had molecular weights of less than 500 g/mol (MW 500 g/mol). For BBB permeability, all of the substances showed negative results. However, in the case of human intestinal absorption, positive results were found for all of the three selected compounds.

## 4. Discussion

The disc diffusion assay enabled the screening of honey for the inhibition of the growth of pathogens as affected by floral source and concentration. The antibacterial action of several varieties of honey was found to be related to their DNA degrading activity, which was mediated by coupling the action of hydrogen peroxide and phenolics with radical scavenging activity [60]. Numerous studies have been published on the antimicrobial activities of honey relying on a variety of different ways of acting [1]. The test pathogen and, to a lesser extent, the strain of each pathogen, all played a role in determining the existence and diameter of the zones of inhibition. In the present study, the antimicrobial activities of two honey samples were evaluated against two pathogenic bacteria isolated from spoiled milk. Morphological and biochemical studies confirmed the isolated strains as *Bacillus cereus* and *Listeria monocytogenes*. The antibacterial properties of honey serve as the foundation for a variety of applications including medications and the preservation of both raw and processed foods [61]. When tested against *Bacillus cereus* and *Listeria monocytogenes* at a concentration of 50 mg/disc, stingless honey had the largest diameter in the inhibitory zone. In comparison to the other two, Bacillus cereus showed greater susceptibility to both honey samples. According to their botanical sources, honey’s physiochemical qualities vary, and they are essential to its antibacterial activity [62,63]. More specifically, the sorts of flowers from which bees collect nectar affect the antibacterial action of honey [64]. As a result, we also tested a few physicochemical properties for the two samples of honey. The way bacterial strains react to samples of honey also influence the antibacterial action. Compared to the Gram-negative bacteria, the Gram-positive bacteria are more resistant to essential oils [65]. The honey samples’ pH was very acidic, suggesting that they would work better against bacteria whose ideal pH is higher than 7.5. Stingless honey had a higher water content than the other sample and showed stronger effectiveness against the tested microorganisms. Therefore, honey samples with lower concentrations had a greater antibacterial action. This conclusion is comparable to those looked into by Laallam et al. in 2015 [62]. Additionally, it was found that higher proline and reducing sugar contents were positively correlated with elevated antibacterial activity.

The most important bacterial virulence factors are pore-forming toxins. Hemolysin II, the target protein of the bacterium *Bacillus cereus*, is known to be harmful. N. Rudenko et al. denoted that monoclonal antibody HlyIIC-20 has the ability to suppress HlyII hemolysis (6d5z) [66,67]. Therefore, this novel protein might be an antibiotic target because its pore-forming activity causes apoptosis [68]. Here, molecular docking studies were performed to analyze a protein complex’s kinetics of interaction [56]. Our research findings were paired with an in silico technique to find a potential honey ingredient that may be used to make antibiotics to fight bacteria. Here, 2,4-dihydroxy-2,5-dimethyl-3(2H)-furan 3-one, beta.-D-glucopyranose,1,6-anhydro, and 4H-pyran-4-one,2,3-dihydro-3,5-dihydroxy-6-methy showed the most effective results against the virulence protein hemolysin II of the bacteria. The top compound 2,4-dihydroxy-2,5-dimethyl-3(2H)-furan 3-one, beta is one of the recognized honey compounds with antibacterial properties [69,70,71,72,73]. However, the top three complexes made non-bonded connections with the protein’s active site. The compound’s ability to bind to the protein’s active site indicates that it is effective at inhibiting the protein [74]. Additionally, a variety of data gleaned from simulated trajectories for docked structures indicated rigid conformations and the less flexible nature of the complexes. The root mean square deviations for the C-alpha atoms of the docked complexes, the radius of gyrations, the solvent accessible surface area, the root mean square fluctuations, and the hydrogen bond of the systems are all connected to the binding stability of the complexes. The top three candidates also met the Lipinski rule of five, which states that a compound’s molecular weight should be less than 500 g/mol and that the number of H-bond acceptors and donors should not exceed ten and five, respectively. Additionally, each of them showed a high capacity for gastrointestinal absorption. The standards for being a viable drug candidate are these [56]. As a result, it can be said that such substances have the potential to be effective against microorganisms and that they could be employed to create future antibiotics. This study may offer a new window into the production of antibiotics from natural sources to control foodborne pathogenic bacteria because there have been no other studies that have studied the antibacterial activity of honey samples using a combination of in vitro and in silico work.

## 5. Conclusions 

Antibiotic-resistant bacteria that can cause foodborne sickness and other diseases have been linked to the development of antibiotics. The influence of various kinds of honey on the development of bacteria varies. Each organism reacts differently to different types of honey, and honey’s antibacterial properties are the result of several distinct components working together. Stingless honey outperformed the other two samples in terms of its antibacterial efficacy against the two identified foodborne pathogenic bacteria. Moderate antibacterial activity was detected in sting honey. The major compounds of honey were tested against the virulence protein of *Bacillus Cerecesus* and three potential candidates (2,4-dihydroxy-2,5-dimethyl 3(2H)-furan-3-one (furan), beta.-D-glucopyranose,1,6-anhydro, and 4H-pyran-4-one,2,3-dihydro 3,5-dihydroxy-6-methyl) were identified, which represent an interesting alternative option to treat persistent wounds infected with this bacterium.

## Figures and Tables

**Table 1:** The morphological and biochemical properties of Isolate A and Isolate B.

	Isolate A	Isolate B
Gram Staining	+	+
Motility test	+	+
Indole test	-	-
Urease hydrolysis test	+	-
TSI	+	-
Methyl red test	-	+
Citrate test	+	-
Catalase test	+	+
Eosin methylene blue (EMB) agar test	-	+
Mannitol agar test	-	-
Starch hydrolysis test	+	-

**Table 2:** The antibiotic sensitivity test for the detection of the resistance pattern of *Bacillus cereus* and *Listeria monocytogenes*.

Antibiotics	*Bacillus Cereus*		*Listeria Monocytogenes*	
	Zone of Inhibition (mm)	Resistance Pattern	Zone of Inhibition (mm)	Resistance Pattern
Penicillin G	7 ± 0.52	R	8 ± 0.34	R
Ampicillin	7.5 ± 0.48	R	7 ± 0.65	R
Amoxicillin,	6 ± 0.67	R	17 ± 0.76	S
Ciprofloxacin	16 ± 0.43	S	8 ± 0.71	R
Chloramphenicol	16 ± 0.56	S	9 ± 0.81	R
Erythromycin	18 ± 0.72	S	8 ± 0.29	R
Gentamycin	18 ± 0.59	S	6 ± 0.34	R
Tetracycline	19 ± 0.39	S	13 ± 0.32	I

N.B. Resistant =< 10 mm; Intermediate = 10–15 mm; Susceptible => 15 mm.

**Table 3:** The physicochemical parameters of the honey samples.

Parameters	Stingless Honey	Sting Honey
Color	Blackish	Light Black
pH	4.35 ± 0.52	4.56 ± 0.62
Water content (%)	18 ± 0.76	16 ± 0.67
Hydroxy methyl furfural (mg/kg)	123.05 ± 1.54	94.76 ± 2.01
Total sugars (%)	87.34 ± 1.34	81.09 ± 2.09
Reducing sugars (%)	77.84 ± 1.32	67.10 ± 1.82
Proline (mg/kg)	833.02 ± 2.89	783.76 ± 2.91

**Table 4:** The antimicrobial activity of stingless and sting honey against *Bacillus cereus* and *Listeria monocytogenes*.

Bacteria	Dose (mg/Disc)	Stingless Honey	Sting Honey
		Zone of Inhibition (mm)	Zone of Inhibition (mm)
*Bacillus cereus*	20	10 ± 0.67	7.5 ± 0.60
	30	11 ± 0.76	9 ± 0.39
	40	13 ± 0.54	9.5 ± 0.40
	50	14 ± 0.89	12 ± 0.52
	Gentamycin (10 µg)	15 ± 0.91	16 ± 0.74
*Listeria monocytogenes*	20	7.5 ± 0.29	7 ± 0.41
	30	8.5 ± 0.76	8 ± 0.42
	40	10 ± 0.89	9.5 ± 0.48
	50	12 ± 0.54	11 ± 0.51
	Gentamycin (10 µg)	13 ± 0.49	14 ± 0.21

**Table 5:** The protein–ligand interaction and identification of binding residues.

SI NO	Compound	Docking Score	Hydrogen Bond	
			Residues	Distance (A°)
1.	2,4-dihydroxy-2,5-dimethyl 3(2H)-furan-3-one (furan)	–5.4	PHE-57 ASN-2 ASN-2	5.17 3.56 2.46
2.	beta.-D-glucopyranose,1,6-anhydro	–5.2	PHE-57 ASN-2	5.39 3.61
3.	4H-pyran-4-one,2,3-dihydro 3,5-dihydroxy-6-methyl	–4.8	ASN-2	3.27

**Table 6:** The pharmacological assessment of the screened hit ligand molecules.

Parameters	2,4-Dihydroxy-2,5-Dimethyl 3(2H)-Furan-3-One (Furan)	Beta.-D-Glucopyranose,1,6-Anhydro	4H-Pyran-4-One,2,3-Dihydro 3,5-Dihydroxy-6-Methyl
Molecular weight	144.13 g/mol	162.14 g/mol	144.13 g/mol
Num. H-bond acceptors	4	5	4
Num. H-bond donors	2	3	2
TPSA (S)	66.76 Å²	79.15 Å²	66.76 Å²
BBB permeability	No		No
Human intestinal absorption	High	High	High
P-glycoprotein substrate	No	No	No
Lipinski rule of five	Yes; 0 violation	Yes; 0 violation	Yes; 0 violation

## Data Availability

All data generated or analyzed during this study are included in this published article (and its supplementary information file).

## Supplementary Materials

The following are available online at https://www.mdpi.com/article/10.3390/molecules27196536/s1, Figure S1: Morphological test; Mortality test (A). Biochemical test of isolate A and isolate B. Urease hydrolysis test (B), Methyl red test (C), TSI test (D), Simmons citrate test (E) Catalase test (F), EMB agar test (G), Mannitol salt agar test (H), Bismuth sulfide agar test (I) and Starch agar test (J). Table S1: Molecular docking of Stingless honey compounds and the bacterial targeted protein (6d5z). Table S2: Molecular docking of sting honey compounds and the bacterial targeted protein (6d5z).
