# Supplementary material for: Biological Efficacy of Compounds from Stingless Honey and Sting Honey against Two Pathogenic Bacteria: An In Vitro and In Silico Study
Source: Molecules. 2022 Oct 3;27(19):6536. doi: 10.3390/molecules27196536 (PMC9570921; doi:10.3390/molecules27196536)
Supplement: Supplementary file 1 [file molecules-27-06536-s001.zip › molecules-1929518-supplementary.pdf]

Biological efficacy of compounds from stingless honey and sting honey against two pathogenic bacteria: an in vitro and in silico study

Shirmin Islam†,[1], Joy Pramanik†,[1], Suvro Biswas[1], Moniruzzaman[1], Jui Biswas[1], Akhtar-E-Ekram[1], Shahriar Zaman[1], Salah Uddin[1] and Abu Saleh*,[1], Sabry Hassan[2]

[1]Microbiology Laboratory, Department of Genetic Engineering and Biotechnology, University of Rajshahi, Rajshahi-6205, Bangladesh
[2]Biology Department, College of Science, Taif University, P.O. Box 11099, Taif 21944, Saudi Arabia
†Equal contribution.
* Correspondence: authors' Email: saleh@ru.ac.bd

Table S1: Molecular docking of Stingless honey compounds and the bacterial targeted protein (6d5z).

SL No	Compound Name	CID	MW	Docking Score
1.	Furfural	7362	96.08	-4.0
2.	Furfuryl Alcohol	7361	98.10	-3.9
3.	2-Hydroxy-2-cyclopenten1-one	82674	98.10	-4.2
4.	Methyl succinic anhydride	20051	114.10	-4.5
5.	2-Furancarboxaldehyde, 5-methyl	12097	110.11	-4.1
6.	2,4-Dihydroxy-2,5-dimethyl-3(2H)-furan 3-one	538757	144.12	-5.4
7.	furan-2,5-dicarboxaldehyde	69980	124.09	-4.1
8.	4H-Pyran-4-one,2,3-dihydro-3,5-dihydroxy-6-methy	119838	144.12	-4.8
9.	4H-Pyran-4-one,3,5-dihydroxy-2-methyl	70627	142.11	-4.8
10.	5-Formyl-2-furfurylmethanoate	58489131	140.14	-4.6
11.	2-Furancarboxaldehyde,5-(hydroxymethyl) (furan derivative)	237332	126.11	-4.3
12.	beta.<br>-D-Glucopyranose,1,6-anhydro	13037722	162.14	-5.2

Table S2: Molecular docking of sting honey compounds and the bacterial targeted protein (6d5z).

SL. No	Compound Name	CID	MW	Docking Score
1.	Furfuryl Alcohol	7361	98.1g/mol	-3.9
2.	2-Hydroxy-2-cyclopenten 1-one	62752	126.15g/mol	-4.8
3.	2,4-Dihydroxy-2,5-dimethyl 3(2H)-furan-3-one (furan)	538757	144.12 g/mol	-5.4
4.	4H-Pyran-4-one,2,3-dihydro 3,5-dihydroxy-6-methyl	119838	144.12 g/mol	-4.8
5.	2-Furancarboxaldehyde, 5	24354	100.12 g/mol	-3.7
6.	Decycltetraglycol	526715	334.5g/mol	-4.4
7.	Tetra ethylene glycol monododecyl ether	78933	362.5 g/mol	-4.1

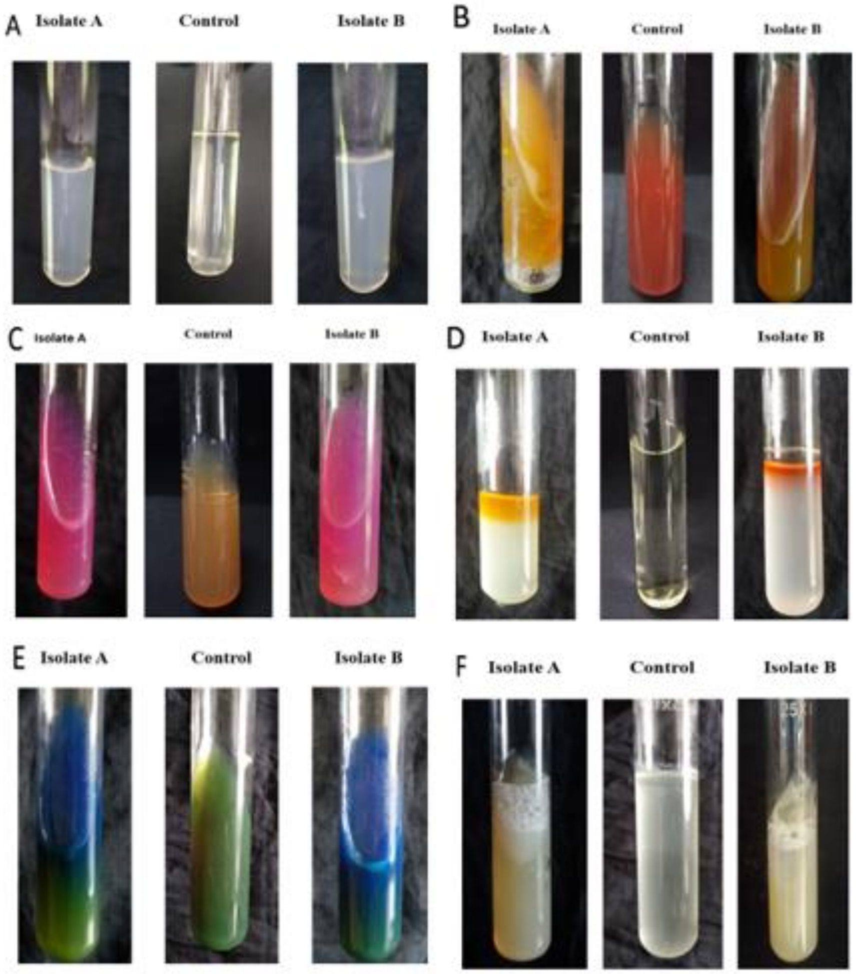

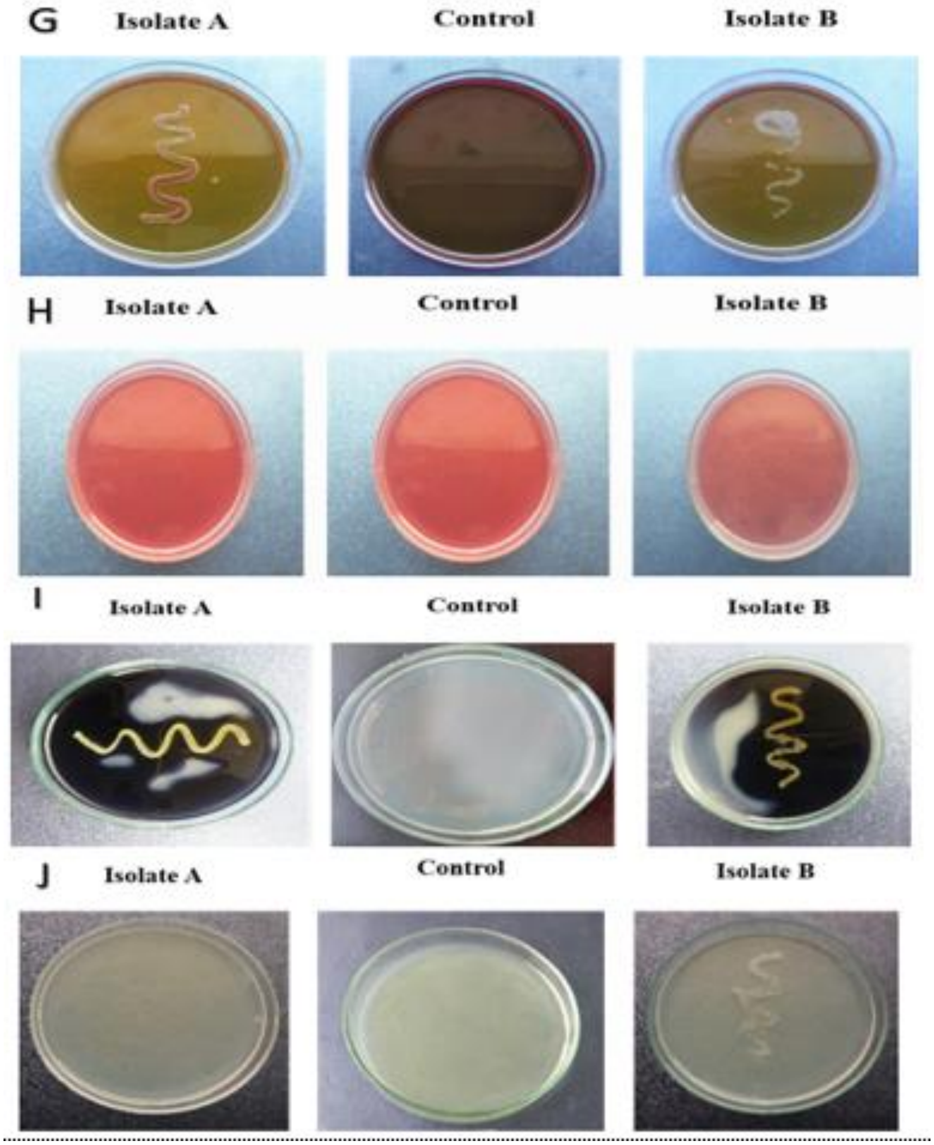

Fig S1. Morphological test; Mortality test (A). Biochemical test of isolate A and isolate B. Urease hydrolysis test (B), Methyl red test (C),TSI test (D) ,Simmons citrate test (E) Catalase test (F), EMB agar (G), Mannitol salt agar (H) , Bismuth sulfide agar test (I) and Starch agar test (J).
